# RNase 7 and Th cytokines synergistically increase the secretion of interleukin-6 from keratinocytes

**DOI:** 10.1038/s41598-025-04403-8

**Published:** 2025-06-03

**Authors:** Shruti Chopra, Janine Siegl, Verena Kopfnagel, Sylvia Dreyer, Franziska Rademacher, Juliane Lokau, Christoph Garbers, Jürgen Harder, Thomas Werfel, Katinka Döhner

**Affiliations:** 1 Department of Dermatology and Allergy, Hannover Medical School, Hannover, Germany; 2 Department of Dermatology, Quincke Research Center, Kiel University, Kiel, Germany; 3 Institute of Clinical Biochemistry, Hannover Medical School, Hannover, Germany; 4 Cluster of Excellence RESIST (EXC 2155), Hannover Medical School, Hannover, Germany; 5 Institute of International Animal Health/One Health, Friedrich-Loeffler-Institut, Greifswald, Germany

**Keywords:** RNase 7, IL-6, IL-24, IL-17, IFN-γ, Inflammatory skin diseases, Immunology, Diseases, Pathogenesis

## Abstract

Epidermal keratinocytes are a major source of antimicrobial peptides and proteins. RNase 7 (R7) exhibits antimicrobial and immunomodulatory activities and is upregulated in the lesional skin of atopic dermatitis and psoriasis patients. Here, we performed mRNA microarray analysis to study the influence of R7 on gene expression in human keratinocytes. R7 induced several genes encoding proteins potentially involved in skin integrity and inflammation, including IL-24. Since IL-24 is involved in the pathogenesis of inflammatory skin diseases and since it is induced by IL-6, we further characterized the effect of R7 on IL-6 and IL-24 expression and secretion. R7 increased the release of the cytokines IL-6 and IL-24 by keratinocytes. Using a function-blocking antibody against IL-6 we showed that the R7-induced IL-24 secretion was triggered by IL-6. IL-6 released from R7-stimulated keratinocytes induced STAT3 signaling in target keratinocytes, suggesting a potential pathway resulting in IL-24 induction. Pre-stimulation with IL-17 or IFN-γ, cytokines involved in the pathogenesis of psoriasis, further enhanced R7-induced IL-6 secretion. R7 increased IL-6 secretion mainly from undifferentiated keratinocytes, suggesting that in the epidermis, R7 released from the stratum granulosum may induce expression and secretion of cytokines from basal undifferentiated keratinocytes. In conclusion, our data show that R7 enhances the release of IL-6 in undifferentiated primary keratinocytes, which may have consequences for skin inflammation, defense against pathogens, and wound healing.

**Supplementary Information:**

The online version contains supplementary material available at 10.1038/s41598-025-04403-8.

## Introduction

Epidermal keratinocytes constitute a structural part of the innate immune system. They secrete antimicrobial peptides and proteins (AMPs) with antifungal, antibacterial, antiviral, and immunomodulatory properties, including RNase 7 (R7) of the RNase A family^1–5^.

While the 14.5 kDa protein R7 is constitutively expressed and secreted at relatively high levels, its expression can be further increased by cytokines, growth factors and microbial factors^4^. As a major AMP of human skin, R7 contributes to protect skin from bacterial colonization and infection^6–11^. Together with low levels of costimulatory DNA, R7 induces an antiviral response that protects keratinocytes against herpes simplex virus type 1 (HSV-1) infection^12,13^. At higher but still physiological concentrations, R7 also restricts HSV-1 infection without the addition of costimulatory DNA by interfering with an early step in the infectious cycle^14^.

Atopic dermatitis (AD), one of the most common inflammatory skin diseases, is characterized by an impaired skin barrier, an imbalance between type 1 T helper cells (Th1) and type 2 T helper cells (Th2), and drastic shifts in the skin microbiota^15,16^. There is increasing evidence that the function of AMPs may be disturbed in AD, which may promote microbial dysbiosis and colonization with pathogenic bacteria, in particular *Staphylococcus (S.) aureus*^17,18^. In this regard, we recently reported that increased amounts of DNA and the ribonuclease inhibitor inhibit the antibacterial activity of R7 in AD, which may promote *S. aureus* growth^19,20^.

The acute phase of AD is associated with increased production of Th2 cytokines, in particular interleukin-4 (IL-4), IL-13, and IL-31. We have shown that R7 reduces the expression of Th2 cytokines by human Th2 cells and that this effect is diminished in AD patients^21^. The chronic phase of AD is characterized by upregulation of Th1 cytokines, particularly interferon-γ (IFNγ)^22,23^. IFN-γ activates keratinocytes and induces surface molecules, chemokines and cytokines, including IL-6^24–28^. At the same time, IFN-γ stimulates apoptosis of keratinocytes^29^.

Psoriasis is an inflammatory skin disease characterized by excessive proliferation and impaired differentiation of keratinocytes in psoriatic plaques^30^. Psoriasis may be initiated when AMPs and self-genetic material released from damaged cells form complexes that can activate myeloid and plasmacytoid dendritic cells, which then secrete tumor necrosis factor α (TNF-α), IL-23, and IL-12, resulting in the differentiation and proliferation of Th17 and Th1 cells. Activated Th17 and Th1 cells secrete IL-17 and IFN-γ and maintain psoriatic inflammation. The Th17 cytokines IL-17, IL-21, and IL-22 activate keratinocyte proliferation in the epidermis, and keratinocytes actively participate in the inflammatory cascade by secreting IL-1β, IL-6, TNF-α, and AMPs^30^.

Depending on the context, IL-6 can exert pro- or anti-inflammatory functions^31^. IL-6 links innate and adaptive immunity to regulate antimicrobial defense. It controls the survival of Th1 and Th2 cells by enhancing the effects of other lymphokines and functions as a cytokine commitment cue for the proliferation of Th17 and Th22 cells^31,32^. In addition, IL-6 activates the production of Th2 cytokines in CD4 + T cells. The single nucleotide polymorphisms (SNPs) rs2069837 in *IL6* and rs2228145 and rs12133641 in *IL6R* are significantly associated with AD, and blocking IL-6 signaling with an antibody against its receptor can alleviate AD symptoms^33–36^. On the other hand, therapeutic IL-6R antibodies can cause dermatitis, and inborn errors in *IL6R* cause immunodeficiency, atopy including atopic dermatitis, elevated IgE levels, and abnormal inflammatory responses^37–39^. Moreover, the rs2228145 SNP in *IL6R* renders IL-6R more susceptible to proteolysis thereby increasing the amount of soluble IL-6R and decreasing the amount of membrane-bound IL-6R^40,41^. These data suggest a complex role for IL-6 signaling in AD pathogenesis that requires further investigation^41^.

IL-24 is a member of the IL-20 family of cytokines and is produced by various cell types, including CD4 + T cells, and keratinocytes^42^. IL-24 has anti-tumor activity and is required for optimal wound healing but also contributes to the pathogenesis of AD, psoriasis, arthritis, and inflammatory bowel diseases. Its expression is induced by several cytokines, including IL-1β, TNF-α, IFN-γ, IL-4, IL-6, IL-17A, IL-22, and IL-31 as well as by UV light^26,42–44^. IL-24 downregulates filaggrin, a protein required for epidermal barrier integrity, and upregulates the inflammatory mediators IL-8, cyclooxygenase-2, and matrix metalloproteinase-1, indicating that it is involved in the positive feedback control of epidermal inflammation^26,42,43,45^.

Here, we used mRNA microarray analysis to identify several genes involved in inflammation and skin integrity whose expression is regulated by R7. We further analyzed how R7 and Th cytokines influence the expression and secretion of one of our hits from the screen, IL-24, as well as IL-6, which triggers IL-24 release.

## Results

### R7 induced the expression and release of the cytokines IL-6 and IL-24 by keratinocytes

To examine the effect of R7 on gene expression, we stimulated human primary keratinocytes (HPK) with R7 and performed an mRNA microarray assay. R7 upregulated the expression of several transcripts (Table 1), including transcripts encoding proteins potentially involved in skin integrity (KRTAP2-3, keratin-associated protein 2–3; MMP3 and 10, matrix metallopeptidase 3 and 10) and inflammation (CXCL3, C-X-C motif chemokine ligand 3; IL13RA2, IL-13 receptor α2; IL-24; SLCO2 A1, solute carrier organic anion transporter family member 2 A1). IL6 was not classified as an up-hit in our microarray because R7 induced only a weak upregulation of 1.4-fold and 2.0-fold for donors 1 + 2 and 3 + 4, respectively, with one of two IL6 probes.

Since the Th2 cytokine IL-13 plays a dominant role in the lesional skin of AD patients by enhancing itch sensation and downregulating skin barrier function^46^, we validated the effect of R7 on IL13RA2. Upregulation of IL13RA2, which encodes a decoy receptor for IL-13^46^, was confirmed by qRT-PCR (Supplementary Fig. 1).

**Table 1.**
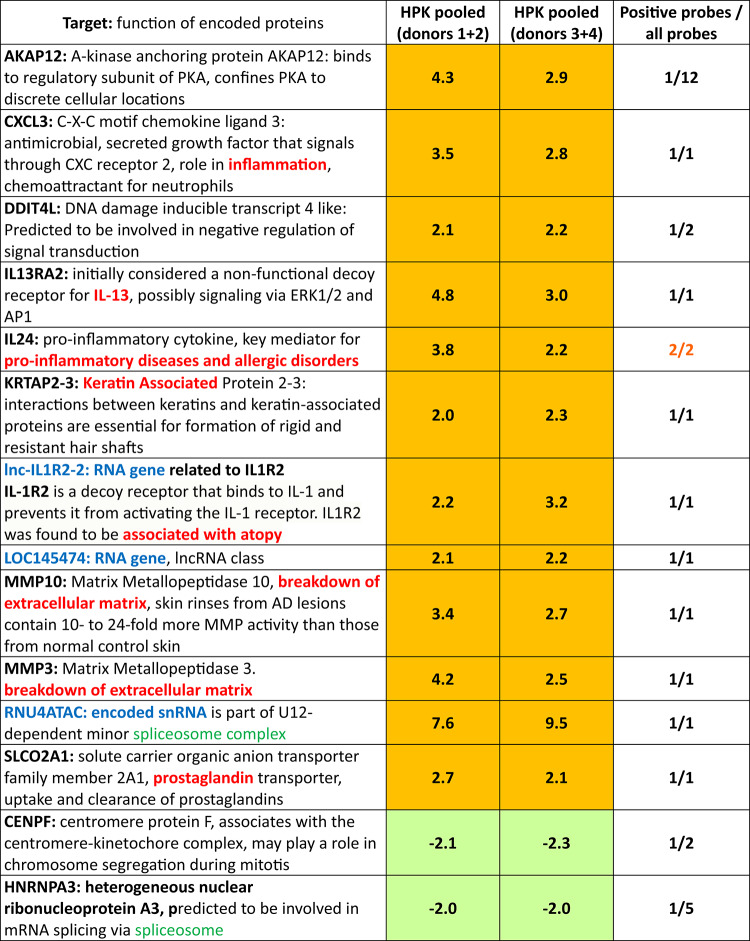
R7 modulated the expression of several genes as identified by an mRNA microarray, followed by filtering for hits that came up in HPK. HPK from four different donors (1-4) were either not stimulated or stimulated with 10 µg/ml R7. After 6 h, cells were harvested and RNA was isolated. The respective samples from two donors (1+2; 3+4) were pooled and the resulting 4 samples (unstimulated, stimulated with R7, respectively for HPK pooled from donors 1+2 or 3+4) were analyzed using a whole human genome oligo-microarray. For upregulated transcripts, the stimulated/unstimulated ratio is shown. For downregulated transcripts, the unstimulated/stimulated * (-1) ratio is indicated. We filtered for hits that were consistently observed after R7 stimulation in both sets of pooled keratinocytes. In the first column, protein-coding targets are shown in black, RNA genes in blue. Involvement in inflammation, skin integrity or atopy is indicated in red, involvement in splicosome function in green. In columns 2-3, uphits with a stimulation of > 2-fold are shown in orange. Downhits with a> 2-fold reduction are displayed in green. In the fourth column, the same trend with at least two probes is indicated in orange.

**Fig. 1:**
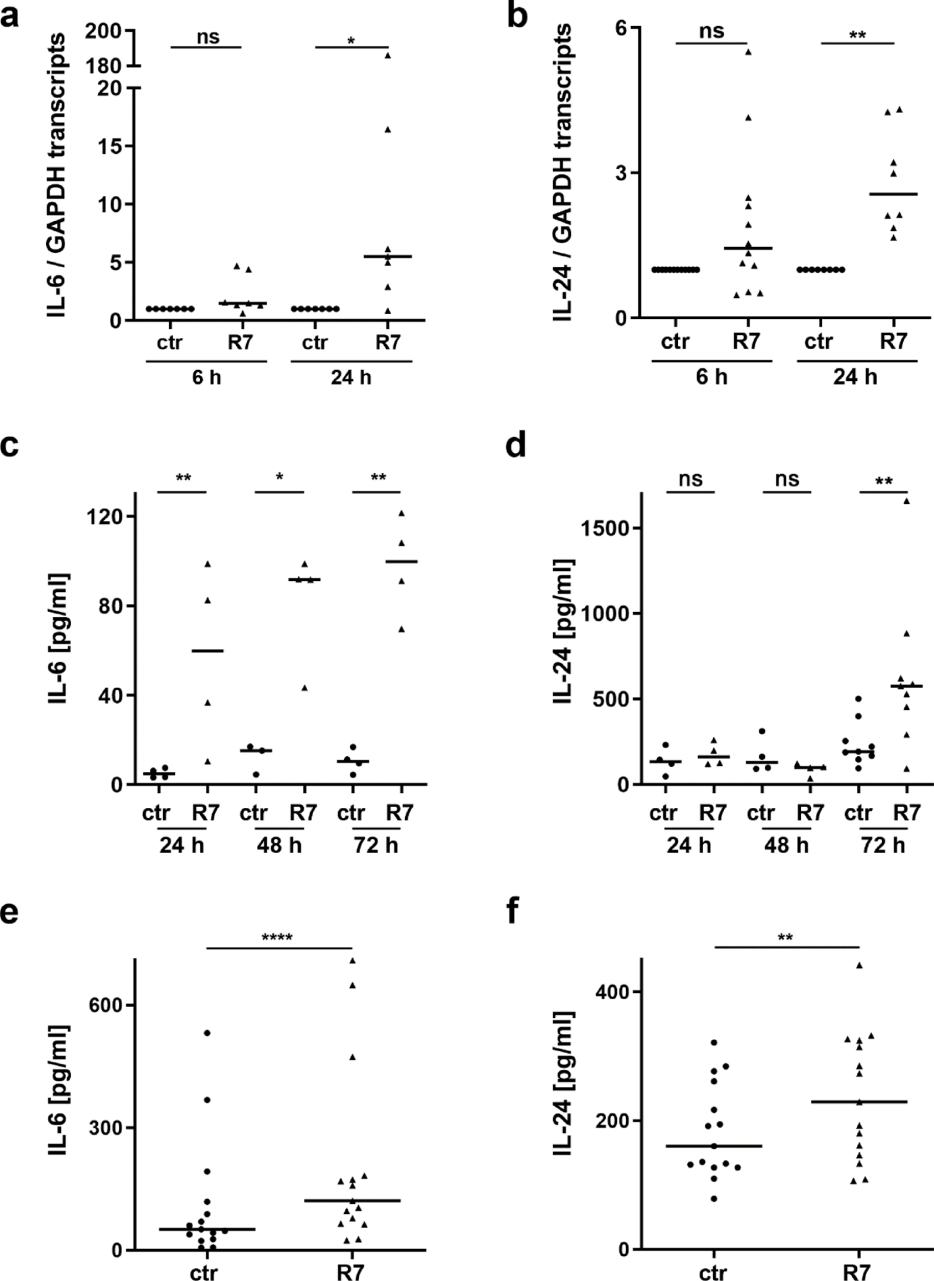
R7 induces expression and secretion of IL-6 and IL-24. (**a**, **b**) 5 x 10^4^ HPK were seeded on a 24-well plate and stimulated with 10 μg/ml R7. The relative expression of IL-6 (n = 7) and IL-24 [n(6 h) = 12, n(24 h) = 8] was analyzed by qRT-PCR. (**c**, **d**) HPK were stimulated with 10 μg/ml R7, and the release of IL-6 (n = 4) and IL-24 (n = 4 for 24 h and 48 h, n = 9 for 72 h) at indicated time points was measured by ELISA. (**e**, **f**) HPK were stimulated with 5 μg/ml R7 for 48 h, and the release of IL-6 (n = 15) and IL-24 (n = 15) was determined by ELISA. Data in (**a**-**b** and **e**-**f**) were statistically analyzed by Wilcoxon matched-pairs signed rank test, data in (**c**,**d**) by paired t test. *p < 0.05, **p < 0.01, ****p < 0.0001, ns, not significant. Bars indicate median; ctr: unstimulated control cells.

To validate the effect of R7 on IL-24 expression and to examine whether it also affects IL-24 secretion and the expression and secretion of IL-6, which has been reported to induce IL-24 expression^26,42^, we performed qRT-PCR and ELISA assays after stimulation of HPK with 10 µg/ml R7 (Fig. 1). IL-6 and IL-24 transcript levels were slightly increased after 6 h and significantly upregulated after 24 h of R7 stimulation (Fig. 1a-b). Accordingly, R7 significantly increased IL-6 release at 24, 48, and 72 h (Fig. 1c). While R7 significantly induced IL-24 release at 72 h, it had no effect at 24–48 h (Fig. 1d). Untreated control (ctr) keratinocytes released higher amounts of IL-24 than IL-6 (Fig. 1c-f), consistent with a high constitutive expression of IL-24 in keratinocytes^47^.

Stimulation experiments (data not shown) with increasing concentrations of R7 for 48 h showed that R7 had little effect on IL-6 secretion from keratinocytes when used at 0.5–1 µg/ml, but a significant effect when used at 5–15 µg/ml. In contrast, the effect of R7 on IL-24 release was strongest when used at 0.5–5 µg/ml and decreased at higher concentrations. Based on these results, we used 5 µg/ml R7 for all subsequent stimulations. Using additional donors, the stimulation of HPK with 5 µg/ml R7 for 48 h significantly increased the release of IL-6 (Fig. 1e) and IL-24 (Fig. 1f). In summary, R7 stimulated the expression and release of the cytokines IL-6 and IL-24 by keratinocytes.

### R7-induced IL-24 secretion and STAT3 signaling depends on IL-6

**Fig. 2:**
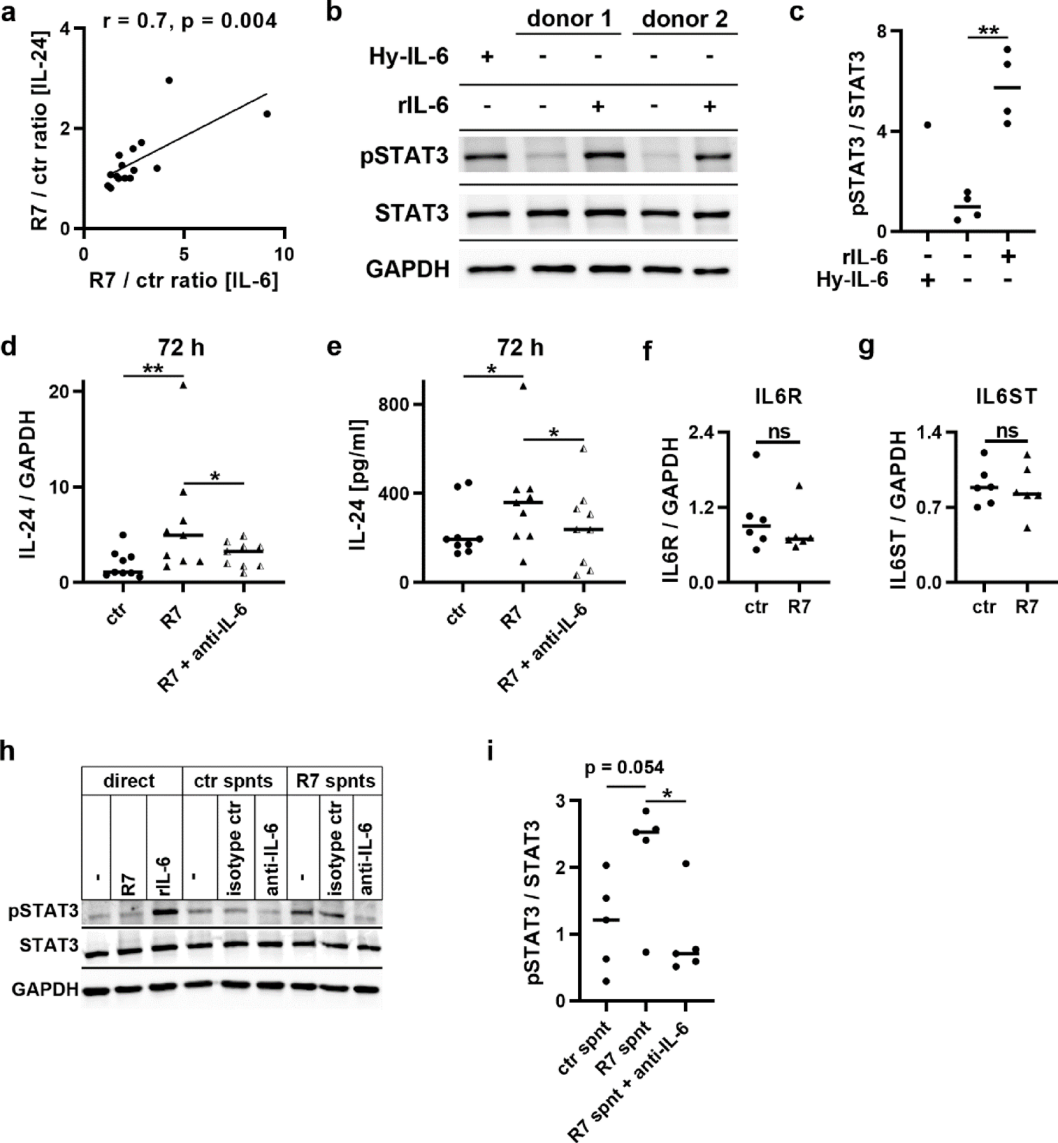
IL-24 secretion correlates with and depends on IL-6 secretion and signaling and IL-6 secreted from R7-stimulated cells activates STAT3 signaling. (**a**) We calculated the ratio of cytokine release from unstimulated and R7-stimulated HPK and plotted the IL-24 ratio against the IL-6 ratio. Statistics: Pearson correlation, significant with p = 0.004, n = 15. (**b**) HPK were starved for 4 h and then stimulated for 15 min with either 10 ng/mL rIL-6, 10 ng/mL Hyper-IL-6 (Hy-IL-6) or left unstimulated. Phosphorylation of STAT3 and total STAT3 protein levels were visualized by immunoblot in comparison to housekeeping GAPDH protein. (**c**) pSTAT3/STAT3 ratios of four HPK donors were quantified and statistical significance was analysed by paired t test; **p < 0.01. Bars indicate median. (**d**, **e**) HPK of 9 donors were seeded on a 24-well plate and were not stimulated (ctr) or stimulated with 5 µg/ml R7 either in the absence or in the presence of 2 µg/ml of a function-blocking antibody against IL-6. After 72 h, we measured IL-24 transcript levels relative to GAPDH transcripts (d) and IL-24 release by ELISA (**e**). Data in (**d**, **e**) was statistically analyzed by Friedman’s test followed by Dunn’s multiple comparisons test. *p < 0.05, **p < 0.01, ns, not significant. Bars indicate median. Relative IL6R (**f**) and IL6ST (**g**) expression of HPK (n = 6) after 48 h without (ctr) or with 5 µg/ml R7 was determined by qRT-PCR and compared by Wilcoxon matched-pairs signed rank (**f**) or paired t test (**g**). Bars indicate median. (**h**) HPK were starved for 4 h and then stimulated for 15 min either directly with 5 µg/ml R7 or 10 ng/ml rIL-6 or with spnts of HPK that had been stimulated for 48 h with 5 µg/ml R7 or left unstimulated, followed by either no treatment or an incubation with 2 µg/ml of a function-blocking IL-6 antibody or an isotype control antibody for 1 h at 37°C. Phosphorylation of STAT3 was analysed by immuno-blotting (**i**) Quantification of pSTAT3 relative to STAT3 bands (n = 5). Statistical analysis was performed with Friedman’s test followed by Dunn’s multiple comparisons test. *p < 0.05. Bars represent median.

IL-6 has been reported to increase IL-24 transcript and protein levels in keratinocytes^26,48^. Since R7 upregulated the expression and release of both IL-6 and IL-24 (Fig. 1), we examined whether IL-24 secretion correlated with IL-6 release (Fig. 2a). We first determined the increase in the concentration of secreted IL-6 or IL-24 after stimulation with R7. We calculated the R7/ctr ratio of IL-6 secretion for all keratinocyte donors by dividing the IL-6 secretion from R7-stimulated cells by the IL-6 secretion from unstimulated ctr cells. The ctr/R7 ratio of IL-24 secretion was calculated accordingly. The R7/ctr ratio for IL-24 release was then plotted against the R7/ctr ratio for IL-6 release (Fig. 2a). There was a strong and significant correlation between the R7/ctr ratios of IL-24 and IL-6 release (Fig. 2a; Pearson’s correlation coefficient *r* = 0.7, *p* = 0.004), suggesting that IL-6 released upon R7 stimulation may bind to IL-6 receptors on keratinocytes and trigger IL-24 expression and release.

IL-6 plays an important role in skin homeostasis and wound repair, and IL-6 and its receptors are produced by human keratinocytes^49,50^. IL-6 activates its target cells via binding to the non-signaling IL-6 receptor (IL-6R), followed by recruitment of the signal-transducing β-receptor gp130 encoded by IL6ST, activation of cytoplasmic tyrosine kinases and subsequent phosphorylation of signal transducer and activator of transcription (STAT) proteins^31^. While gp130 is ubiquitously expressed, only a limited number of cell types express IL-6R and are thus direct targets of IL-6^51^. To test whether the HPK used in this study were responsive to IL-6, we first serum-starved them for 4 h and then stimulated them for 15 min with either 10 ng/ml recombinant IL-6 (rIL-6) or hyper-IL-6 (Hy-IL-6), a fusion protein of IL-6 and the soluble IL-6R, or left them unstimulated. Stimulation with rIL-6 or with the positive control Hyper-IL-6 triggered STAT3 phosphorylation (Fig. 2b-c), confirming that the HPK used in this study express both IL-6 receptors that enable them to signal via STAT3 in response to IL-6 stimulation.

To test whether IL-24 release was dependent on IL-6 secretion, we added a function-blocking antibody against IL-6 during R7 stimulation (Fig. 2d-e). Without anti-IL-6 antibody, R7 significantly increased IL-24 transcripts (Fig. 2d) and IL-24 secretion (Fig. 2e) compared to untreated control cells. The anti-IL-6 antibody significantly, but not completely, reduced the R7-induced increase in IL-24 expression (Fig. 2d) and secretion (Fig. 2e). This suggests that the upregulation of IL-24 expression and secretion was mostly a consequence of the R7-induced upregulation of IL-6 secretion. R7 had no significant effect on the expression of the IL-6 receptor complex formed by IL6R and IL6ST (Fig. 2f-g). Stimulating cells for 15 min with R7 did not directly induce phosphorylation of STAT3 (Fig. 2h). However, incubating keratinocytes for 15 min with supernatants (spnts) of cells that had been stimulated for 48 h with R7 resulted in phosphorylation of STAT3, which was inhibited by an anti-IL-6 but not an isotype control antibody (Fig. 2h-i).

In summary, we showed that R7-induced IL-24 secretion correlated with and was dependent on IL-6 secretion, and that IL-6 secreted from R7-stimulated cells can activate STAT3 signaling in keratinocytes.

### Effect of R7 on IL-6 and IL-24 release from keratinocytes after pre-stimulation with Th1, Th2 or Th17 cytokines

**Fig. 3:**
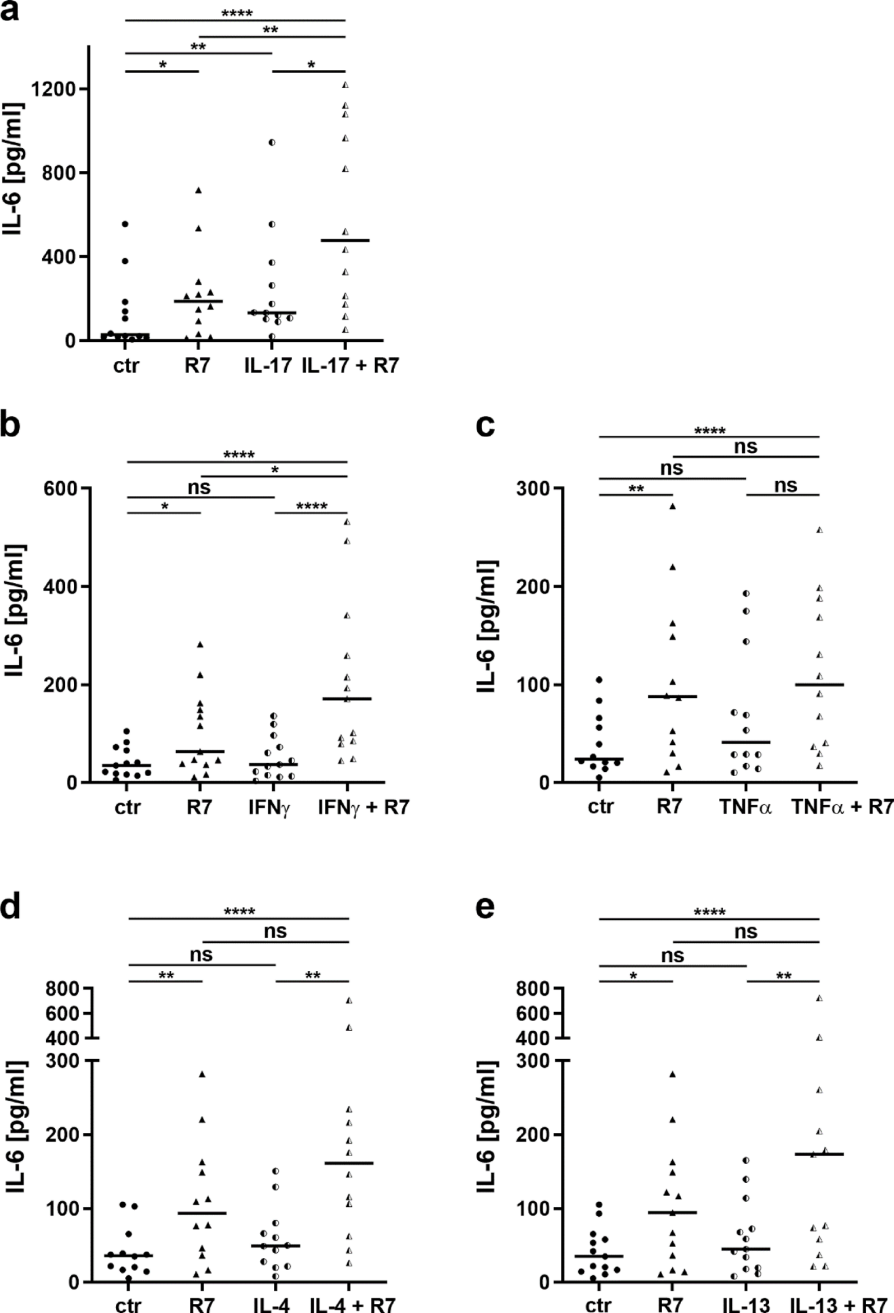
Prestimulation with IL-17 or IFN-γ enhances R7-induced IL-6 release. IL-6 release from HPK pre-stimulated for 24 h with (**a**) 10 ng/ml IL-17 (n = 12), (**b**) 10 ng/ml IFN-γ (n = 13), or (**c**) 50 ng/ml TNF-α (n = 12), (**d**) 50 ng/ml IL-4 (n = 12), or (**e**) 50 ng/ml IL-13 (n = 13), followed by stimulation with 5 μg/ml R7 for another 48 h. Cytokine release was determined by ELISA. Data were statistically analysed by Friedman’s test followed by Dunn’s multiple comparisons test. *p < 0.05,**p < 0.01, ***p < 0.001, ****p < 0.0001, ns, not significant. Bars indicate median; ctr: unstimulated control cells.

Next, we addressed the influence of the disease-specific cytokines of psoriasis (Th1, Th17) or AD (Th2) on the R7-triggered release of IL-6. To this end, we prestimulated HPK with the respective cytokines of Th17 cells (IL-17), Th1 cells (IFN-γ, TNF-α), or Th2 cells (IL-4, IL-13) and subsequently stimulated with R7 (Fig. 3).

To investigate the specific effect of these cytokines on HPK, we first stimulated cells with IL-17, IFN-γ, TNF-α, IL-4 or IL-13 without additional stimulation with R7. Stimulation with 10 ng/ml IL-17 significantly induced IL-6 release (Fig. 3a). In contrast, stimulation with 10 ng/ml IFN-γ, 50 ng/ml TNF-α, 50 ng/ml IL-4 or 50 ng/ml IL-13 had no significant effect on IL-6 release (Fig. 3b-e). Stimulation with 5 µg/ml R7 for 48 h reproducibly and significantly stimulated IL-6 release (Fig. 3) as observed before (Fig. 1).

Stimulation of HPK with 5 µg/ml R7 for 48 h after pre-stimulation with IL-17, IFN-γ, TNF-α, IL-4 or IL-13 significantly increased IL-6 release compared to unstimulated HPK (Fig. 3). Prestimulation with IL-17 (Fig. 3a) or IFN-γ (Fig. 3b), but not with TNF-α (Fig. 3c), significantly enhanced the stimulatory effect of R7 alone. The Th2 cytokines IL-4 and IL-13 tended to enhance the R7-induced upregulation of IL-6 secretion, but their effect was not significant (Fig. 3d-e).

While stimulation of HPK with IL-17 (Supplementary Fig. 2a), IFN-γ, or TNF-α alone (not shown) had no significant effect on IL-24 secretion, IL-4 and IL-13 significantly upregulated IL-24 secretion (Supplementary Fig. 2b-c). After 48 h, R7 alone tended to increase IL-24 secretion (Supplementary Fig. 2a). Prestimulation with IL-17, IL-4, or IL-13 followed by R7 stimulation significantly increased IL-24 release when compared to unstimulated control cells (Supplementary Fig. 2a-c), while IFN-γ or TNF-α in combination with R7 had no such effect (not shown).

In conclusion, our data suggest that R7-stimulated release of IL-6 is further enhanced by IL-17 or IFN-γ, cytokines that contribute to the pathogenesis of psoriasis, and that the release of IL-24 is upregulated by IL-4 or IL-13, cytokines that drive the pathogenesis of AD, or a combination of IL-17 and R7.

### R7 increased IL-6 secretion from differentiated keratinocytes slightly, but not significantly

Keratinocytes differentiate as they migrate from the stratum basale to the stratum corneum during their development^52^. In vitro, keratinocyte differentiation can be induced by Ca^2+ 53–55^.

**Fig. 4:**
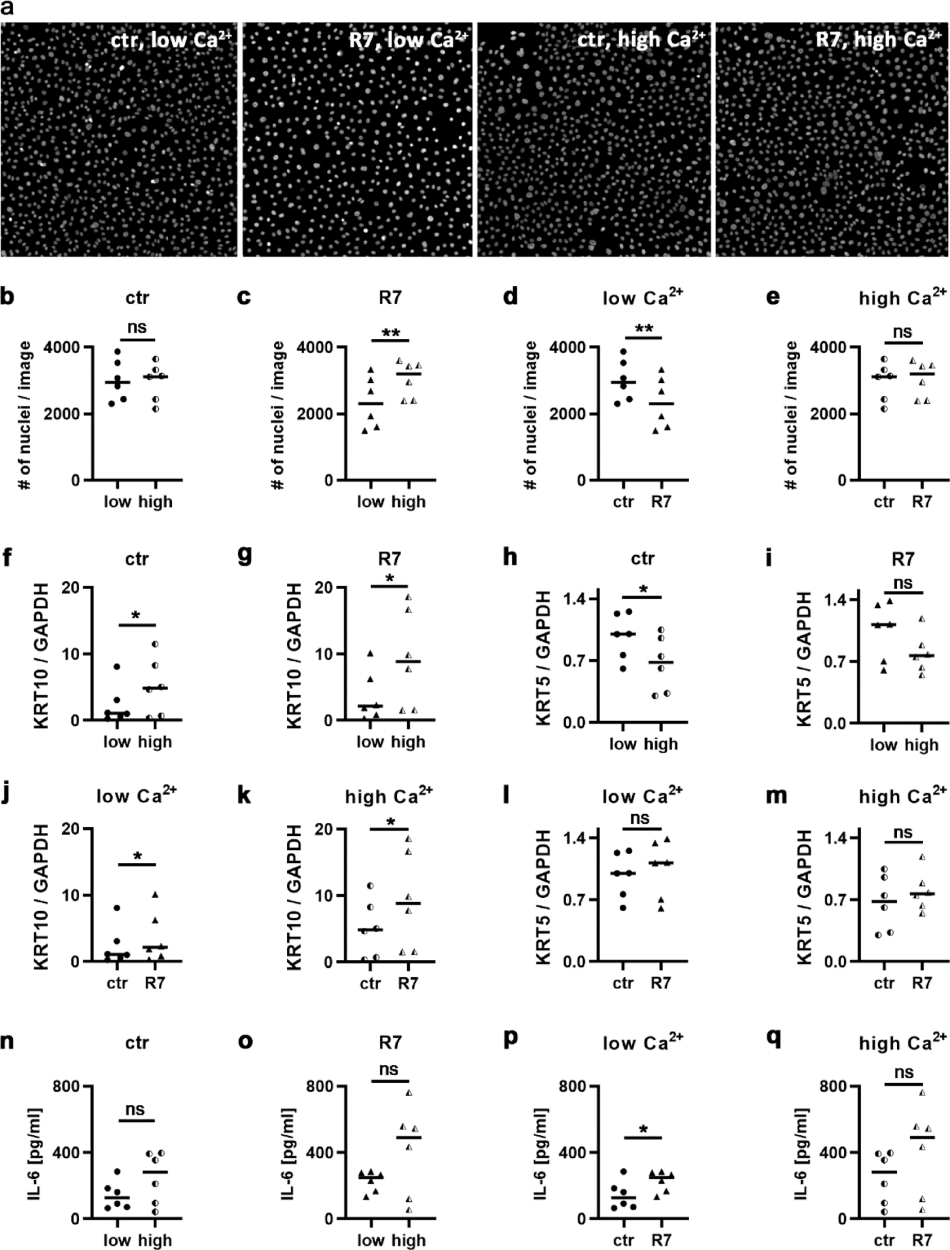
R7 increases IL-6 secretion from differentiated keratinocytes slightly, but not significantly. HPK were cultured for 24 h in media with either 0.06 or 1.46 mM CaCl_2_ and then stimulated for another 48 h with 5 μg/ml R7 in the continued presence of either 0.06 or 1.46 mM CaCl_2_. Afterwards, (**a**) cell densities were assessed by automated microscopy after DAPI staining and (**b**-**e**) quantified by CellProfiler. (**f-m**) Relative expression of KRT10 and KRT5 was determined by qRT-PCR. (**n**-**q**) IL-6 release was measured by ELISA. Data (n = 6) were statistically analyzed using paired t test (**b**-**e**, **g**-**i**,**k**-**m**, **n**-**q**), or Wilcoxon matched-pairs signed rank test (**f**, **j**). **p < 0.01,*p < 0.05, ns, not significant. Please note, that (**f**-**i**) and (**j**-**m**) are derived from the same data set. Bars indicate median; ctr: unstimulated control cells.

We induced differentiation by elevating the CaCl_2_ concentration in complete medium from 0.06 to 1.46 mM. After 24 h, we stimulated the cells in stimulation medium for another 48 h with 5 µg/ml R7 in the continued presence of either 0.06 or 1.46 mM CaCl_2_ (Fig. 4). Increasing Ca^2+^ levels in unstimulated cells had no effect on cell density (Fig. 4a-b). Under low but not high Ca^2+^ conditions, R7 reduced cell density when compared to unstimulated cells (Fig. 4a-e).

Increasing CaCl_2_ levels induced differentiation in ctr and R7-stimulated HPK as revealed by increased keratin 10 (KRT10) and reduced KRT5 levels (Fig. 4f-i). R7 increased KRT10 levels in undifferentiated and differentiated keratinocytes but did not affect the expression of KRT5, a marker for basal keratinocytes (Fig. 4j-m). Increasing Ca^2+^ levels tended to increase IL-6 secretion in both unstimulated and R7-stimulated keratinocytes (Fig. 4n-o). Consistent with previous experiments, R7 significantly stimulated IL-6 release from undifferentiated HPK (Fig. 4p). In differentiated keratinocytes, it increased IL-6 secretion slightly, but not significantly (Fig. 4q).

## Discussion

### R7 influences the expression of several genes involved in skin integrity and inflammation

The expression and secretion of R7 is increased in the skin of patients with inflammatory skin diseases^4,56–59^. Here, we performed an mRNA microarray analysis that identified IL-24 as one of the genes upregulated by R7 even without addition of costimulatory DNA. In addition, R7 upregulated the expression of several other genes encoding proteins potentially involved in skin integrity (KRTAP2-3, keratin-associated protein 2–3; MMP3 and 10, matrix metallopeptidase 3 and 10) and inflammation (CXCL3, C-X-C motif chemokine ligand 3; IL13RA2, IL-13 receptor α2; IL-24; SLCO2 A1, solute carrier organic anion transporter family member 2 A1). Interestingly, R7 induced the expression of DNA damage inducible transcript 4 like (DDIT4L; Table 1), while its paralog DNA damage inducible transcript 4 (DDIT4) has previously been shown to be downregulated by R7 in a human liver cancer cell line^60^. In addition, R7 and IL24 are upregulated upon knockdown of DDIT4 in a human oral squamous carcinoma cell line^61^. How R7 exerts its effects on keratinocytes requires further investigation. R7 is expressed and secreted by keratinocytes and belongs to the RNase A superfamily^4,5^. While other members of this family such as RNase A, RNase 3 (eosinophil cationic protein, ECP) and RNase 5 (angiogenin) are internalized by various pathways^62–65^, there is, however, no evidence for R7 internalization by mammalian cells. R7 binds to the extracellular domain of the receptor tyrosine kinase c-ros oncogene 1 (ROS1) and triggers ROS1 signaling as well as phosphorylation of several targets including STAT2 and STAT6 and changes in gene expression in hepatocellular carcinoma cells indicating that ROS1 can function as receptor for R7^60^. However, ROS1 is mainly expressed in the male reproductive system and the lung and only at low levels in the skin (The Human Protein Atlas; Tissue expression of ROS1 - Summary - The Human Protein Atlas) with virtual no expression in basal and suprabasal keratinocytes at the single cell level (The Human Protein Atlas; Single cell type - ROS1 - The Human Protein Atlas). These data suggest that in keratinocytes other receptors besides ROS1 might mediate the effects of R7.

We confirmed that R7 induced the expression and release of IL-24 and IL-6, which was previously reported to upregulate IL-24 expression^26,42,48^. Since IL-6 and IL-24 can contribute to inflammation, defense against cutaneous infections, and wound healing^31,42,47,66–69^, we propose that R7 promotes defense against cutaneous infections and wound healing by stimulating their expression and secretion.

### R7 triggers the release of IL-6, which subsequently induces IL-24

Compared to its effect on IL-6, the effect of R7 on IL-24 was delayed, and the stimulatory effect of R7 on IL-24 secretion correlated with its effect on IL-6 release. Human keratinocytes were responsive to IL-6, and interference with IL-6 signaling inhibited the R7-triggered induction of IL-24 and reduced IL-24 secretion. This indicates that IL-24 was mostly induced by IL-6, which was in turn upregulated by R7, consistent with an induction of IL-24 by IL-6 as previously reported^26,42,48,70^. Stimulating human keratinocytes with 25 ng/ml IL-6 for 24 h induces IL-24 transcripts and IL-24 secretion^26^. Incubation of murine keratinocytes with 10 ng/ml IL-6 for 24 h induces IL-24 expression at the mRNA level, and this upregulation is strongly enhanced in suppressor of cytokine signaling 3-deficient mouse keratinocytes, indicating that it depends on STAT3 signaling^48^. In human oral keratinocytes, 10 ng/ml IL-6 induces the IL24 transcripts in a bimodal manner with higher levels after 3 and 6 h, lower levels after 12 h and again higher levels at 24 h^70^. All these experiments were performed with IL-6 concentrations (10–25 ng/ml) that were by a factor of 100 higher than those induced by R7 in our assays (on average 100 pg/ml). This bimodal response, together with the lower IL-6 concentrations present in our assays, makes it difficult to compare the delay in IL-24 release that we observed with these reports. However, given the lower IL-6 levels present during our experiments, a delay of 24–48 h appears to be within a reasonable range.

R7 did not upregulate the expression of IL6R or IL6ST, nor did it directly induce STAT3 phosphorylation after 15 min of incubation. However, spnts from cells that had been treated for 48 h with R7 induced STAT3 phosphorylation in keratinocytes after 15 min of incubation. This was most likely mediated by IL-6 secreted into the spnt as STAT3 phosphorylation was inhibited by a function-blocking anti-IL-6 antibody. Since IL-6-induced upregulation of IL-24 also depends on STAT3 signaling^48^, R7 may induce IL-6 secretion, which then triggers STAT3 signaling resulting in increased expression and release of IL-24.

Both R7 and IL-6 are upregulated after *Aspergillus flavus* infection of human corneal epithelial cells and after infection of control or thermally wounded skin equivalents with methicillin-resistant *S. aureus*, but it was not studied, whether the upregulation of IL-6 was triggered by increased R7 levels or both were induced by the same triggering factors^71,72^. In bladder epithelial cells, R7 reduces infection with uropathogenic *Escherichia coli* and the upregulation of IL-6 expression after infection with these bacteria under high glucose conditions by regulating the JAK/STAT signaling pathway^73^. This suggests that the effect of R7 on IL-6 may depend on the specific cell type and the presence of other triggering factors.

### Th cytokines modulate the stimulatory effect of R7 on IL-6 and IL-24 release

AD or psoriatic skin with increased R7 secretion is also exposed to higher Th cytokine levels^4^. We therefore tested whether the effects of R7 and Th cytokines on IL-6 and IL-24 release are interdependent.

IL-17A, IFN-γ, and IL-4 were previously reported to upregulate the expression of IL-6^25–27^. Consistently, we found that IL-17 alone significantly increased IL-6 release. However, neither IL-4 nor IFN-γ alone induced IL-6 secretion in our hands. These differences could be due to a different readout (mRNA versus secreted protein) or differences in cytokine concentrations or stimulation times. IL-17 and IFN-γ, which play a critical role in the pathogenesis of psoriasis, significantly enhanced the R7-triggered IL-6 secretion, and the Th2 cytokines IL-4 and IL-13, which play a critical role in the pathogenesis of AD, tended to enhance the stimulatory effect of R7 on IL-6 secretion.

In contrast to a previous report^26^, IL-17 alone did not significantly upregulate IL-24 release in our hands. Consistent with previous reports^26,43,47^, we found that the Th2 cytokines IL-4 and IL-13 increased the release of the pro-inflammatory cytokine IL-24. While R7 alone only slightly increased IL-24 secretion at 48 h, it significantly induced IL-24 release when combined with the Th cytokine IL-17, and tended to increase the effects of IL-4 and IL-13.

These findings suggest that high expression and secretion of R7, as found in AD or psoriatic skin^4,56–59^, together with elevated levels of IL-17 or IFN-γ, may drive the release of IL-6, thereby contributing to inflammation, protection against cutaneous pathogens, and wound healing. In addition, elevated IL-24 levels may contribute to inflammation by increasing the expression and secretion of IL-8 and matrix metallopeptidase 1, as well as the expression of cyclooxygenase-2 and the secretion of prostaglandin E2, which are important inflammatory mediators in human keratinocytes^26^. Interestingly, genes encoding two matrix metallopeptidases (MMP3 and MMP10) and a prostaglandin transporter were also among the up-hits in our screen.

Taken together, IL-17 and IFN-γ significantly enhanced IL-6 induction induced by R7, suggesting that high levels of R7 may promote inflammation thereby protecting lesional AD or psoriatic skin against cutaneous pathogens. On the other hand, R7 has the potential to downregulate the expression of inflammatory mediators and to reduce the secretion of the Th2 cytokines IL-4, IL-5, and IL-13 from Th2 cells and the expression of IL13 in the kidney^4,20,21,74^. Moreover, R7 upregulated the expression of IL13RA2, which encodes a decoy receptor for IL-13. IL-13 signals via two receptors^46^. Binding to its heterodimeric receptor composed of IL-4Rα and IL-13Rα1 induces the activation of downstream Janus kinase 2 and tyrosine kinase 2 and results in activation of STAT3, STAT6, and STAT1. This cascade enhances itch sensation and downregulates the skin barrier function. Alternatively, IL-13 can bind to IL13RA2. This receptor binds IL-13 with high affinity, but its cytoplasmic region lacks signaling motifs. IL-13 responses are increased in cells and mice lacking IL13RA2, indicating that IL13RA2 acts as a decoy receptor for IL-13 and elicits antagonistic activity against IL-13^46^. We hypothesize that an upregulation of IL13RA2 by R7, which is expressed at elevated levels in the lesional skin of AD patients^4,56–59^, may exert protective, anti-inflammatory effects in the context of AD. Thus, R7 may have pro-inflammatory or anti-inflammatory effects depending on the context. Due to its activity against *S. aureus* and HSV-1 together with its ability to enhance IL13RA2 expression and suppress Th2 cytokine secretion^4,12–14,20,21^, R7 is an attractive candidate for the treatment of AD, where these pathogens are major disease drivers and HSV-1 infection can lead to eczema herpeticum. Our studies suggest that its effects on pathogens as well as inflammation should be monitored when developing R7 as a therapeutic.

### R7 tends to upregulate IL-6 release from differentiated keratinocytes

During epidermal differentiation, keratinocytes migrate from the stratum basale to the stratum granulosum, where they are transformed into the stratum corneum. The stratum basale consists of highly proliferative keratinocytes, while the stratum corneum is composed of differentiated, i.e. flattened and denucleated cells^52,75,76^. Here, we analyzed the effect of R7 on keratinocytes in different developmental stages. R7 significantly increased IL-6 release in undifferentiated keratinocytes and also tended to increase IL-6 secretion from differentiated keratinocytes. Without stimulation, differentiated keratinocytes secreted more IL-6 than undifferentiated ones, and the increase by R7 stimulation was not significant. Our data suggest that the ability of keratinocytes to secrete pro-inflammatory cytokines, such as IL-6, increases with differentiation. Since IL-6 inhibits keratinocyte differentiation^77,78^, this could create a feedback loop that further suppresses differentiation. However, R7 significantly upregulated IL-6 secretion in basal keratinocytes, but only tended to increase IL-6 secretion in differentiated keratinocytes. This finding is consistent with stronger IL-6 induction in basal keratinocytes by bacterial stimulation^79^. We hypothesize that an increased release of R7 from the differentiated keratinocytes of AD and psoriasis patients fosters the secretion of IL-6 and IL-24, especially from the undifferentiated keratinocytes of the stratum basale. This may promote inflammatory responses initiated by undifferentiated keratinocytes, while inflammatory responses of suprabasal cells may already be elevated upon the onset of differentiation and may be slightly further increased by R7.

In conclusion, our study shows that R7 upregulates IL-6 and IL-24 expression and secretion, thereby potentially contributing to inflammation, protection against cutaneous infections, and wound healing. In addition, IL-6 release is further enhanced by IL-17 and IFNγ, cytokines involved in the pathogenesis of psoriasis. The immunomodulatory effects of R7 on keratinocytes shown here provide a basis for follow-up studies on the role of R7 against certain bacteria and viruses in AD compared to psoriasis.

## Materials and methods

### Cells and growth conditions

Human primary keratinocytes (HPK) were either purchased from Lonza Group AG (Basel, Switzerland) or obtained from juvenile foreskin^80^ after written informed consent of the patients or their legal representatives and anonymized before further use. This procedure is in accordance with the Declaration of Helsinki and was also approved by The Ethics Committee of the Hannover Medical School (#2603 − 2015). Keratinocytes were cultured in Keratinocyte Growth Medium 2 (PromoCell, Heidelberg, Germany) in the presence (complete medium) or absence (stimulation medium) of 0.125 ng/ml epidermal growth factor (EGF) and 0.33 µg/ml hydrocortisone in an incubator at 37 °C and 5% CO_2_. The medium was changed every two days until the cells reached a confluence of 70–90%. The keratinocytes were then passaged so that the cells could be either further cultured or cryopreserved. Passages two to nine were used for all experiments.

### Oligo-microarray

HPK from four different donors (1–4) were either not stimulated or stimulated with 10 µg/ml R7. After 6 h, cells were harvested and RNA was isolated using a micro RNA kit (Analytik Jena, Jena, Germany) according to the manufacturer´s instructions. The respective samples from two donors (1 + 2; 3 + 4) were pooled and the resulting 4 samples (unstimulated, stimulated with R7, respectively for HPK pooled from donors 1 + 2 or 3 + 4) were analyzed using a whole human genome oligo-microarray (microarray ID: 026652, icosagen, San Francisco, CA, USA).

### Effect of R7 on the expression and release of the cytokines IL-6 and IL-24

HPK were seeded at 5 × 10^4^ cells/well in a 24-well plate and incubated the next day with 400 µl fresh stimulation medium for 24 h, followed by stimulation with 5–10 µg/ml R7 for 6, 24, 48, or 72 h. IL-6 secretion in unstimulated keratinocytes varied considerably, but secretion levels and variability did not depend on the passage number. To test whether IL-24 expression or release depends on IL-6, we blocked IL-6 signaling using a function-blocking IL-6 antibody (2 µg/ml; clone 6708, R&D Systems, Inc., USA), which was renewed after 48 h. RNA was isolated to quantify IL-6 and IL-24 transcripts relative to GAPDH transcripts by qRT-PCR, and the release of IL-6 and IL-24 was measured by ELISA.

### Influence of Th cytokines on R7-induced IL-6 release

HPK were seeded in a 24-well plate one day before stimulation. The next day, the cells were incubated with 400 µl fresh stimulation medium for 3.5 h, followed by pre-stimulation with Th1, Th2, or Th17 cytokines. In this setup, two wells were used for each of the cytokines IL-4 (50 ng/ml), IL-13 (50 ng/ml), IL-17 (10 ng/ml), IFN-γ (10 ng/ml) (all from R&D Systems, Inc., USA) and TNF-α (50 ng/ml; BioLegend Inc., USA). After 24 h, 5 µg/ml R7 was added to one of the two wells. After a further incubation of 48 h, we measured IL-6 and IL-24 release by ELISA.

### Effect of R7 on IL-6 release from differentiated keratinocytes

HPK were seeded at 5 × 10^4^ cells/well in a 24-well plate. Next day, we induced differentiation by elevating the CaCl_2_ (PromoCell, Heidelberg, Germany) concentration in the complete medium from 0.06 to 1.46 mM. After 24 h, we stimulated the cells in 400 µl fresh medium with 5 µg/ml R7 in the continued presence of either 0.06 or 1.46 mM CaCl_2_ for another 48 h. Afterwards, we quantified cell densities by automated microscopy, expression of markers of differentiated or basal keratinocytes by qRT-PCR, and IL-6 release by ELISA.

### Quantitative reverse transcription polymerase chain reaction (qRT-PCR)

Quantitative RT-PCR was used to analyze IL-6 and IL-24 expression in HPK without and after R7 stimulation. Total RNA was extracted using the innuPREP DNA/RNA Mini Kit (Analytik Jena GmbH, Jena, Germany). The RNA was then reverse transcribed into cDNA using the RevertAid First Strand cDNA Synthesis Kit (Thermo Fisher Scientific, Inc., USA). Finally, relative IL-6, IL-24, IL13RA2, KRT5, KRT10, IL6R, IL6ST, and GAPDH transcript levels were measured using Qiagen primers for IL-6 (#QT00083720), IL-24 (#QT00059059), IL13RA2 (#QT00042707), KRT5 (#QT00053046), KRT10 (#QT00017045), IL6R (forward: 5’ TCACAACATGGATGGTCAAGGA 3’; reverse: 5’ GTAAGTGCCTGCATGGGGGT 3’), IL6ST (forward: 5’ AGAACAGCATCCAGTGTCACC 3’; reverse: 5’ AGCAAACTTGTGTGTTGCCCATT 3’) and GAPDH (#QT01192646) as reference gene and the QuantiTect SYBR Green PCR Kit to perform the assay with LightCycler SYBR Green I Master (Roche Diagnostics GmbH, Germany). Analysis was performed using LightCycler Relative Quantification Software (Roche Diagnostics GmbH, Germany).

### Enzyme-Linked immunosorbent assay (ELISA)

ELISA was performed according to the manufacturer’s instructions to detect cytokine levels in the cell-free spnts (IL-6, eBioscience, Inc., USA; IL-24, R&D Systems, Inc., USA). The optical densities of the samples were quantified photometrically at 405 nm using a spectrophotometer (FLUOstar Optima). To determine the levels of IL-6 and IL-24, a standard curve was generated for each cytokine (reference range for IL-6: 2–200 pg/ml and IL-24: 62.5–4000 pg/ml).

### Stimulation of keratinocytes with rIL-6, R7, or Spnts of control or R7-stimulated cells and pSTAT3 detection by Immunoblot

HPK were seeded in a 6-well plate 24 h prior to the experiment. Next day, cells were washed three times with PBS and starved in Keratinocyte Growth Medium 2 (PromoCell, Heidelberg, Germany) without any supplements for 4 h. Cells were stimulated for 15 min with 10 ng/ml rIL-6, Hyper-IL-6 (Hy-IL-6), 5 µg/ml R7, or with spnts of HPK that had been stimulated for 48 h with 5 µg/ml R7 in stimulation media or left unstimulated, followed by 1 h incubation at 37 °C with either no antibody, 2 µg/ml of a function-blocking IL-6 antibody, or a mouse IgG1 isotype control antibody (MAB002, R&D systems, MN, USA). Hyper-IL-6 and IL-6 were produced as described previously^81^. Subsequently, cells were lysed on ice with M-PER™ Mammalian Protein Extraction Reagent (Thermo Fisher Scientific, Inc., USA) containing protease inhibitor Pefabloc^®^ SC (Merck) and phosphatase inhibitor PhosSTOP (Roche, Basel, Switzerland). Proteins were isolated and the concentrations were measured using Qubit™ Protein Assay kit (Invitrogen, MA, USA). Equal amounts of protein were loaded on 10% SDS-polyacrylamide gel (Bio-RAD Laboratories, CL, USA) and transferred to a nitrocellulose membrane. Proteins were probed using monoclonal rabbit anti-pSTAT3 antibody (pY705, D3 A7; cat. #9145), monoclonal mouse anti-STAT3 antibody (124H6; cat. #9139) or monoclonal mouse anti-GAPDH antibody (cat. #14 C10; Cell Signaling Technology, MA, USA) at 1:1000 dilution and labeled with secondary anti-rabbit IgG HRP-linked antibody (cat. #7074; Cell Signaling Technology, MA, USA) or anti-mouse IgG HRP-linked antibody (cat. #7076; Cell Signaling Technology, MA, USA) at 1:2000 dilution, respectively. The blots were developed using SignalFire™ Elite ECL Reagent (Cell Signaling Technology, MA, USA), and images were captured using ChemiDoc Imaging Systems (Bio-Rad Laboratories, CL, USA). Restore™ Western Blot Stripping Buffer was used for stripping of the blots. For normalization and quantification, the Image Lab software (Bio-Rad Laboratories, CL, USA) was used. Data are presented as ratio of pSTAT3/STAT3 signals from four to five different donors using GraphPad Prism (version 8.0, GraphPad Software, CA, USA).

### Automated microscopy

5 × 10^4^ HPK were seeded in a 24-well plate and after 24 h incubated in complete medium containing either 0.06 or 1.46 mM CaCl_2_ for an additional 24 h. Afterwards, HPK were stimulated for 48 h in stimulation medium with 5 µg/ml R7 under low or high Ca^2+^ conditions. Cells were fixed with 4% formaldehyde, permeabilized with 0.1% Triton-X-100, stained with DAPI and 20 images per well of a 24-well plate were acquired by automated microscopy [BioTek Cytation 5 Cell Imaging Multimode Reader (Agilent, CA, USA)]). The number of nuclei/image as a proxy for the number of cells per image was quantified by CellProfiler^82^.

### Statistical analysis

GraphPad Prism software versions 5.04 and 7.03 were used for graphing and statistical data analysis. All data were presented with the median. The statistical test was chosen after checking the normal distribution. The following tests were used in the analysis: Kolmogorov-Smirnov test, Shapiro-Wilk test, and D’Agostino-Pearson test.

Paired t test was used for statistical analysis of normally distributed data. Non-normally distributed data were analyzed using the Wilcoxon signed-rank test or the Friedman test followed by Dunn’s multiple comparisons test. A P value < 0.05 was considered statistically significant. A P value < 0.05 is indicated with *, a P value < 0.01 with **, a P value < 0.001 with ***, and a P value < 0.0001 with ****.

## Electronic supplementary material

Below is the link to the electronic supplementary material.

Supplementary Material 1

Supplementary Material 2

Supplementary Material 3

Supplementary Material 4

Supplementary Material 5

Supplementary Material 6

## Data Availability

Original data from this study are available from the corresponding author upon reasonable request.

## Abbreviations

AD: Atopic dermatitis
AMPs: Antimicrobial peptides and proteins
Ctr: Control
ELISA: Enzyme-linked immunosorbent assay
HSV-1: Herpes simplex virus type 1
IFN-γ: Interferon gamma
IFN-β: Interferon beta
IL: Interleukin
IL6ST: Interleukin 6 cytokine family signal transducer
HPK: Human primary keratinocytes
KRT: Keratin
qRT-PCR: Quantitative reverse transcription polymerase chain reaction
R7: RNase 7
ROS1: c-ros oncogene 1
*S. aureus*: *Staphylococcus aureus*
STAT: Signal transducer and activator of transcription
Spnts: Supernatants
Thx: Type x T helper cell
TNF-α: Tumor necrosis factor α
