# Supplementary figures and images for: RNase 7 and Th cytokines synergistically increase the secretion of interleukin-6 from keratinocytes
Source: Sci Rep. 2025 Jun 3;15:19396. doi: 10.1038/s41598-025-04403-8 (PMC12134212; doi:10.1038/s41598-025-04403-8)

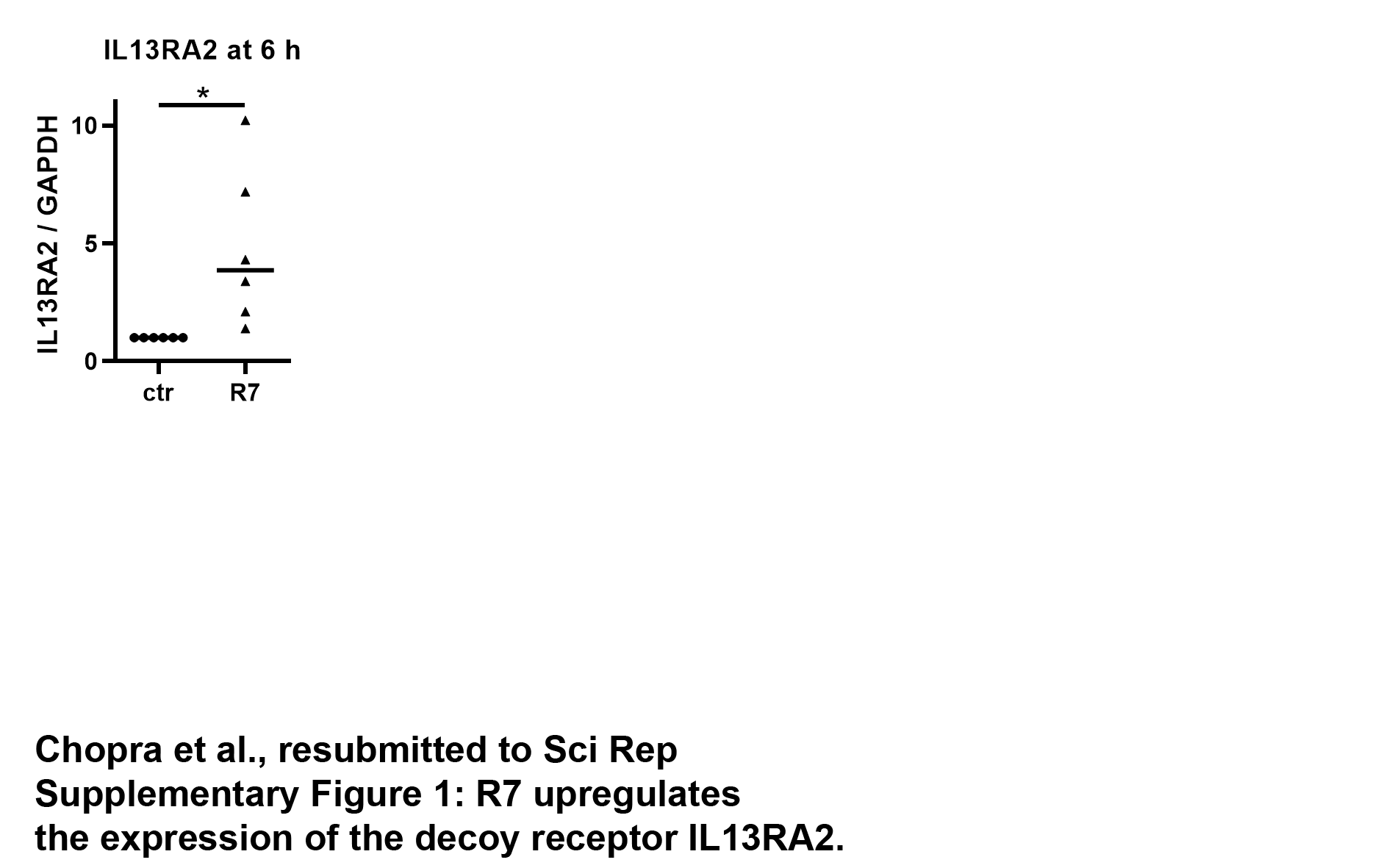

Supplement: Supplementary file 1 — Supplementary Material 1 [file 41598_2025_4403_MOESM1_ESM.tif]

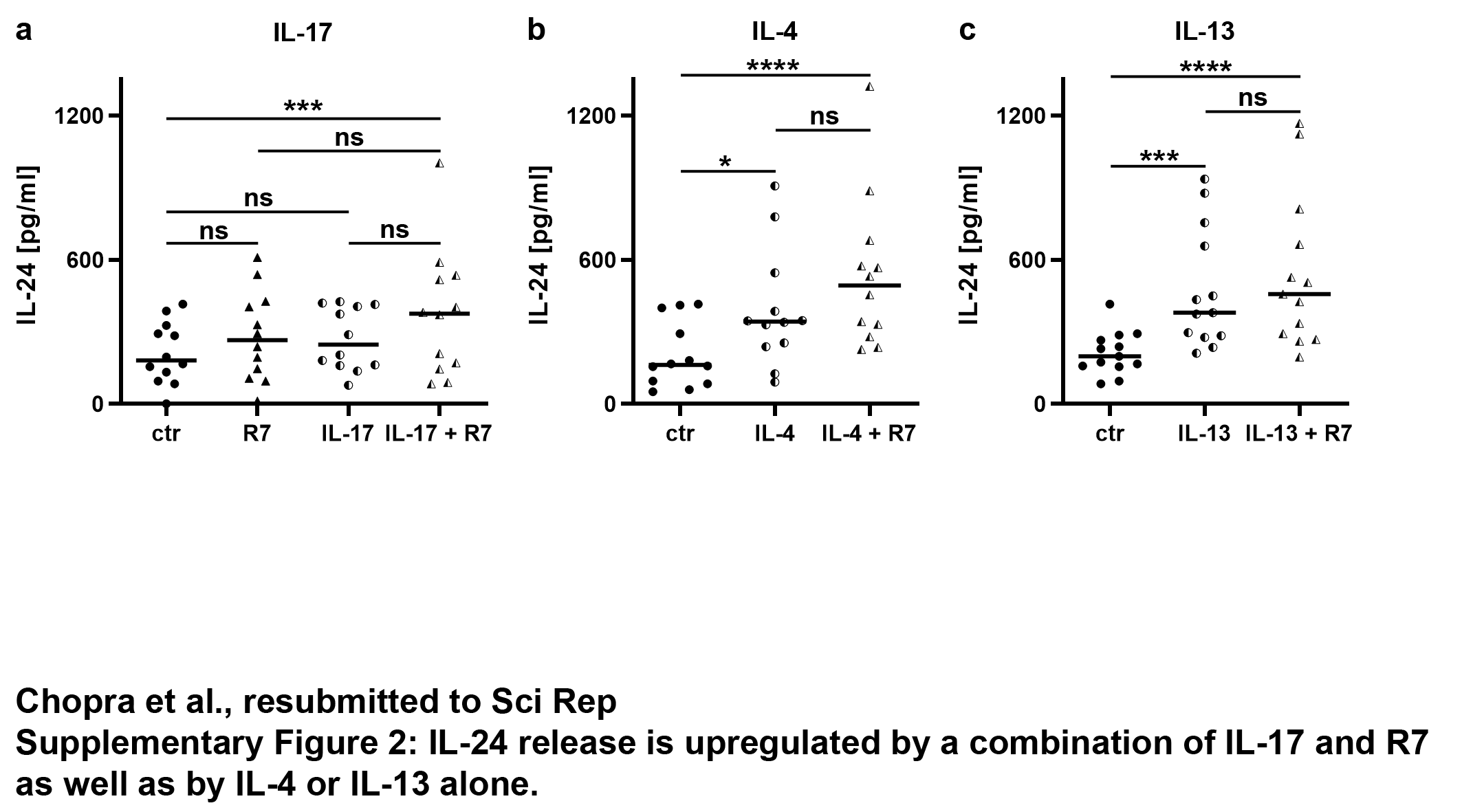

Supplement: Supplementary file 2 — Supplementary Material 2 [file 41598_2025_4403_MOESM2_ESM.tif]

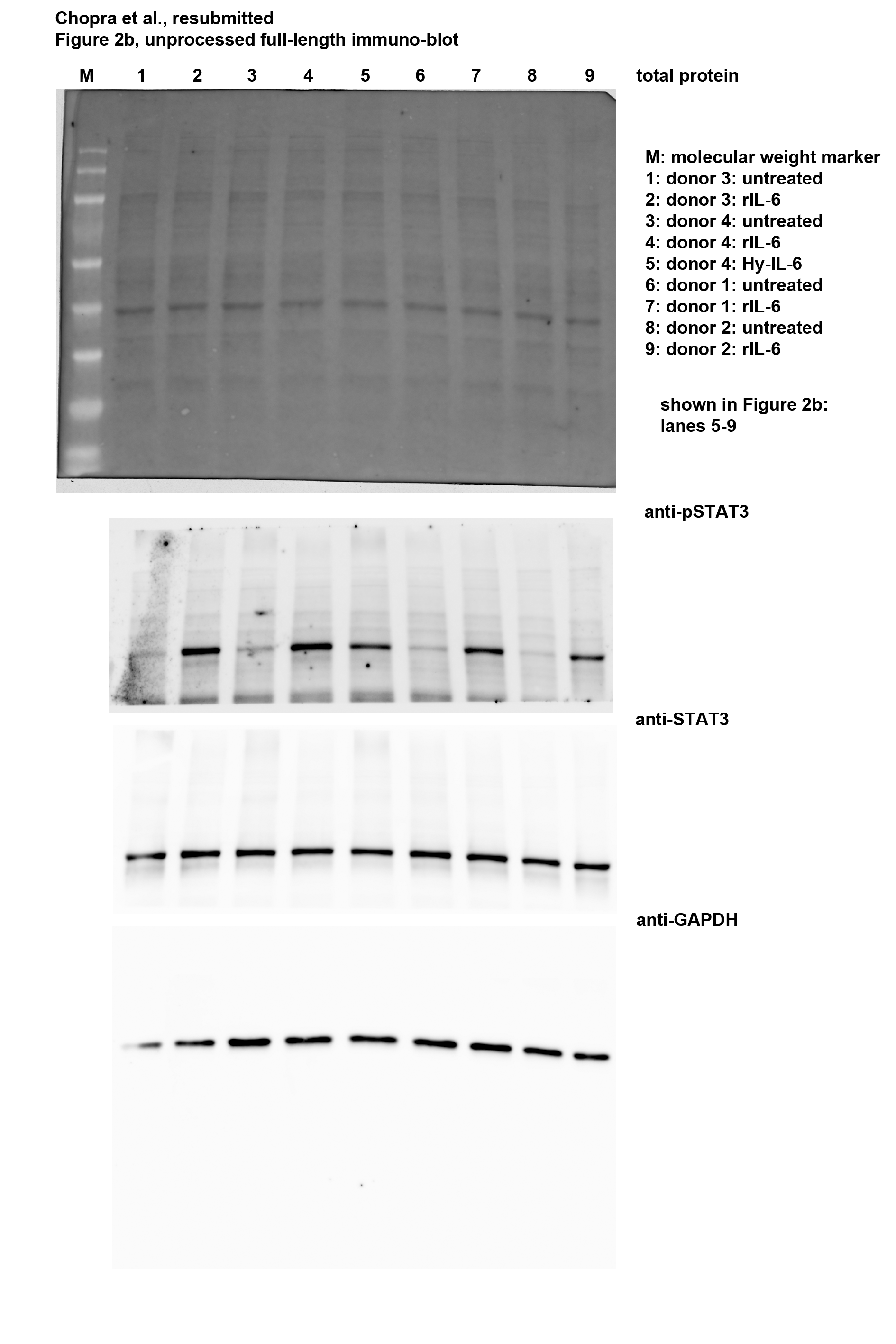

Supplement: Supplementary file 4 — Supplementary Material 4 [file 41598_2025_4403_MOESM4_ESM.tif]

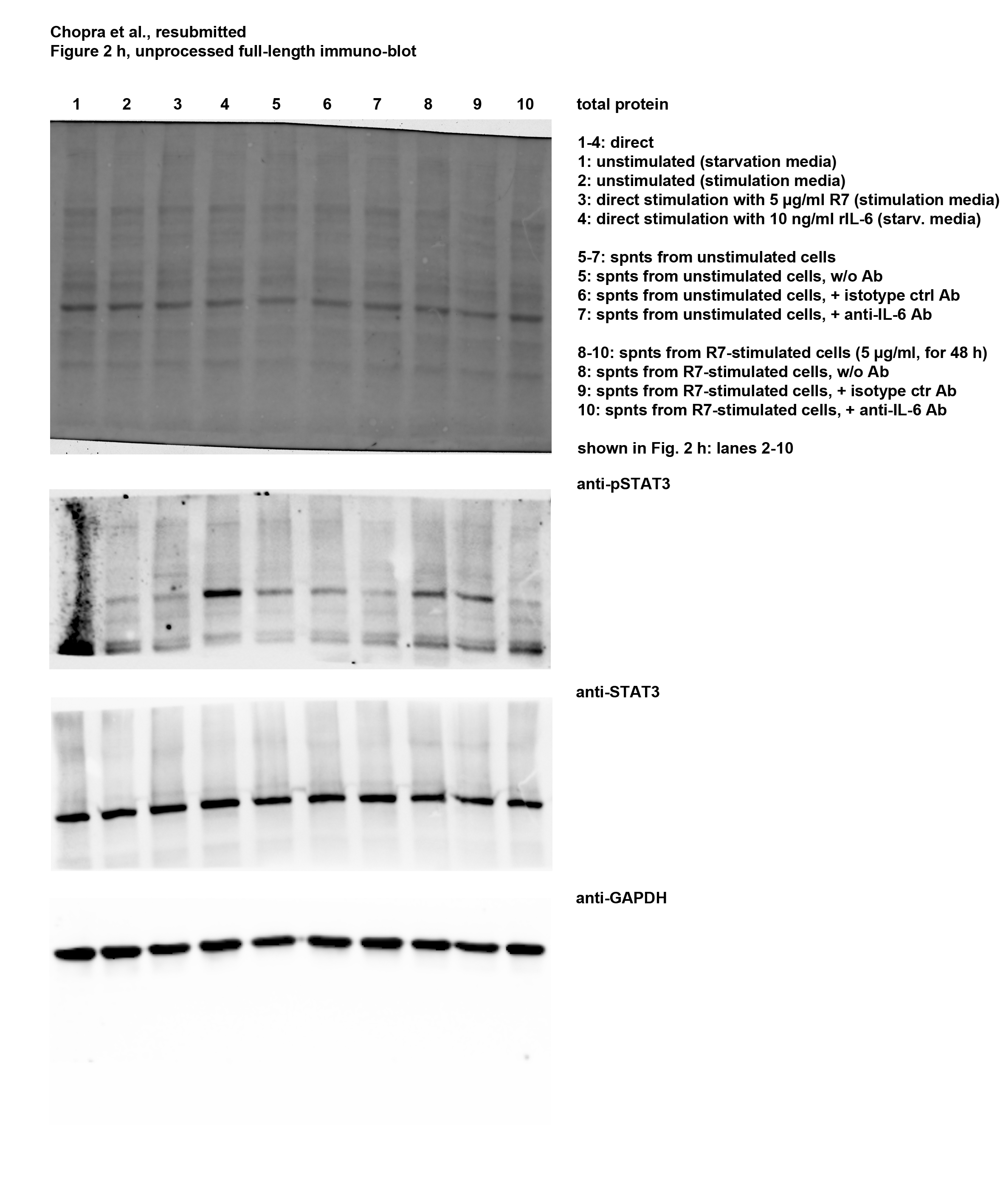

Supplement: Supplementary file 5 — Supplementary Material 5 [file 41598_2025_4403_MOESM5_ESM.tif]

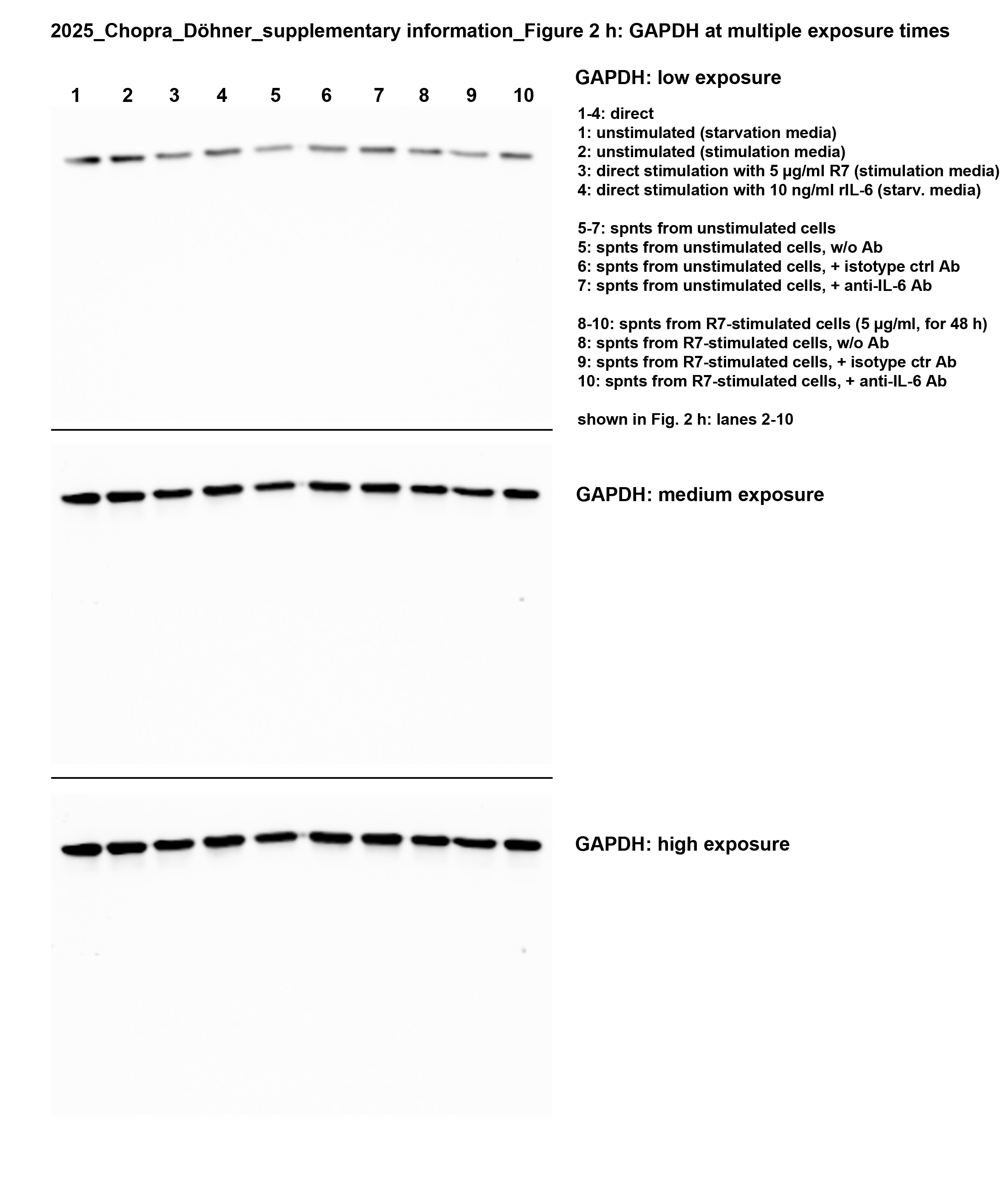

Supplement: Supplementary file 6 — Supplementary Material 6 [file 41598_2025_4403_MOESM6_ESM.tif]
