# Supplementary material for: RNase 7 and Th cytokines synergistically increase the secretion of interleukin-6 from keratinocytes
Source: Sci Rep. 2025 Jun 3;15:19396. doi: 10.1038/s41598-025-04403-8 (PMC12134212; doi:10.1038/s41598-025-04403-8)
Supplement: Supplementary file 3 — Supplementary Material 3 [file 41598_2025_4403_MOESM3_ESM.docx]

**Chopra et al., resubmitted**

**Figure Legends for Supplementary Figures**

**Supplementary Fig. 1: R7 upregulates the expression of the decoy receptor IL13RA2.** 5 x 10^4^ HPK were seeded on a 24-well plate and stimulated for 6 h with 10 µg/ml R7. The relative expression of IL13RA2 was analyzed by qRT-PCR. Data (n = 6) were statistically analyzed by paired t test. * p < 0.05. Bars indicate median; ctr: unstimulated control cells.

**Supplementary Figure 2: IL-24 secretion is upregulated by a combination of IL-17 and R7 as well as by IL-4 or IL-13 alone.** IL-24 release from HPK prestimulated for 24 h with (a) 10 ng/ml IL-17 (n = 12), (b) 50 ng/ml IL-4 (n = 12), or (c) 50 ng/ml IL-13 (n = 13), followed by stimulation with 5 µg/ml R7 for another 48 h. Cytokine release was determined by ELISA. Data were statistically analyzed by Friedman's test followed by Dunn's multiple comparisons test. *p < 0.05, ***p < 0.001, ****p < 0.0001, ns, not significant. Bars indicate median; ctr: unstimulated control cells.
